# Trans-Epithelial Transport, Metabolism, and Biological Activity Assessment of the Multi-Target Lupin Peptide LILPKHSDAD (P5) and Its Metabolite LPKHSDAD (P5-Met)

**DOI:** 10.3390/nu13030863

**Published:** 2021-03-05

**Authors:** Carmen Lammi, Gilda Aiello, Carlotta Bollati, Jianqiang Li, Martina Bartolomei, Giulia Ranaldi, Simonetta Ferruzza, Enrico Mario Alessandro Fassi, Giovanni Grazioso, Yula Sambuy, Anna Arnoldi

**Affiliations:** 1 Department of Pharmaceutical Sciences, University of Milan, 20122 Milan, Italy; gilda.aiello@unimi.it (G.A.); carlotta.bollati@unimi.it (C.B.); jianqiang.li@unimi.it (J.L.); martina.bartolomei@unimi.it (M.B.); enrico.fassi@unimi.it (E.M.A.F.); giovanni.grazioso@unimi.it (G.G.); anna.arnoldi@unimi.it (A.A.); 2 Department of Human Science and Quality of Life Promotion, Telematic University San Raffaele, 00166 Rome, Italy; 3 Food and Nutrition Research Centre, CREA, 00178 Rome, Italy; giulia.ranaldi@crea.gov.it (G.R.); simonetta.ferruzza@crea.gov.it (S.F.); yula.sambuy@crea.gov.it (Y.S.)

**Keywords:** Caco-2 cells, food bioactive peptides, LDLR, PCSK9, trans-epithelial transport

## Abstract

P5 (LILPKHSDAD) is a hypocholesterolemic peptide from lupin protein with a multi-target activity, since it inhibits both 3-hydroxy-3-methylglutaryl coenzyme A reductase (HMGCoAR) and proprotein convertase subtilisin/kexin type-9 (PCSK9). This work shows that, during epithelial transport experiments, the metabolic transformation mediated by intestinal peptidases produces two main detected peptides, ILPKHSDAD (P5-frag) and LPKHSDAD (P5-met), and that both P5 and P5-met are linearly absorbed by differentiated human intestinal Caco-2 cells. Extensive comparative structural, biochemical, and cellular characterizations of P5-met and the parent peptide P5 demonstrate that both peptides have unique characteristics and share the same mechanisms of action. In fact, they exert an intrinsically multi-target behavior being able to regulate cholesterol metabolism by modulating different pathways. The results of this study also highlight the dynamic nature of bioactive peptides that may be modulated by the biological systems they get in contact with.

## 1. Introduction

In addition to their nutritional values, proteins provide numerous health benefits through their ability to modulate one or more targets involved in specific physiological pathways [1,2]. This generally depends on the formation of bioactive peptides that are processed by digestion from the protein sequences and subsequently absorbed at the intestinal level [3,4,5]. These peptides eventually reach the organs where they modulate the target of interest, exerting their biological activity. Indeed, food bioactive peptides are increasingly recognized for their great potential of improving human health and preventing chronic diseases [6].

In this framework, lupin protein hydrolysates, obtained by treating the proteins with pepsin and trypsin, show synergistic hypocholesterolemic effects through the modulation of both 3-hydroxy-3-methylglutaryl coenzyme A reductase (HMGCoAR) and proprotein convertase subtilisin/kexin type-9 (PCSK9) targets [7,8,9,10,11]. In fact, both the peptic and tryptic hydrolysates decrease HMGCoAR activity in vitro, inducing the intracellular low-density lipoprotein receptor (LDLR) pathway, reducing the PCSK9 one, and improving the ability of human hepatic HepG2 cells to uptake low-density lipoproteins (LDL) from the extracellular environment [7,11]. In addition, these lupin hydrolysates impair the protein–protein interaction (PPI) between PCSK9 and LDLR [10]. Although it seems possible that these complementary activities might be due to the synergistic effects of different peptides in the hydrolysates, it cannot be excluded that single peptides endowed of a multi-target inhibitory behavior may be present inside these hydrolysates, which have a very complex composition.

Recently, it has been possible to identify from the peptic hydrolysate peptide P5 (LILPKHSDAD), one of the most potent food peptides capable of inhibiting the PPI between PCSK9 and LDLR [10,12]. A molecular docking analysis has allowed to simulate the effects induced by P5 on this PPI. In fact, the superimposition of P5 on the EGF-A domain of LDLR co-crystallized with PCSK9 (PDB code 4NE9) shows a good overlapping, justifying the P5 inhibitory property [10]. In parallel, an experiment has demonstrated that P5 is able to reduce the catalytic activity of HMGCoAR with an IC_50_ value of 147.2 µM and an in silico investigation has predicted the potential binding mode to the catalytic site of this enzyme [12]. Through the inhibition of the HMGCoAR activity, P5 increases the LDLR protein level on HepG2 cells through the activation of the SREBP-2 transcription factor and, through a down-regulation of HNF-1α, it reduces the PCSK9 protein levels and its secretion in the extracellular environment [12]. This unique synergistic multi-target inhibitory behavior of P5 determines an improved ability of HepG2 cells to uptake extracellular LDL with a final hypocholesterolemic effect. In the peptic protein hydrolysate, P5 stands out also for its favorable transport across the in vitro model of the intestinal barrier provided by differentiated human Caco-2 cells [9].

Considering the very peculiar features of P5, it appeared necessary to get a deeper insight of its bioavailability. Therefore, the first objective of this study was an investigation of the behavior of P5 in the differentiated Caco-2 cell model, focusing the attention either on the transport or the possible concomitant degradation by active peptidases expressed on the apical (AP) membrane, and consequent production of metabolites. For a better understanding of the transport phenomenon, two different conditions were examined, i.e., P5 alone and in mixture with two other lupin peptides, YDFYPSSTKDQQS (P3) and LTFPGSAED (P7), which had already been shown to be transported in the same model system [13]. Interestingly, an abundantly transported metabolite, LPHKSDAD (P5-met), was identified in these experiments. According to the hypothesis that this breakdown peptide may retain a multi-target activity, the second objective of the work was an extensive structural, biochemical, and cellular characterization of P5-met in comparison with P5 as the reference compound.

## 2. Materials and Methods

### 2.1. Chemicals

Dulbecco’s modified Eagle’s medium (DMEM), stable *L*-glutamine, fetal bovine serum (FBS), phosphate buffered saline (PBS), penicillin/streptomycin, chemiluminescent reagent, and 96-well plates were purchased from Euroclone (Milan, Italy). The HMGCoAR assay kit, bovine serum albumin (BSA), Janus Green B, formaldehyde, HCl and H_2_SO_4_ were from Sigma-Aldrich (St. Louis, MO, USA). The antibody against LDLR and the 3,3′,5,5′-tetramethylbenzidine (TMB) substrate were bought from Thermo Fisher Scientific (Waltham, MA, USA). The Quantikine ELISA kit was bought from R&D Systems (Minnneapolis, MN, USA). The LDL-DyLightTM 550 was from Cayman Chemical (Ann Arbor, MI, USA). The CircuLex PCSK9 in vitro binding Assay Kit was from CircuLex (CycLex Co., Nagano, Japan). The peptides (P5, P5-met, P3, and P7) were synthesized by the company GeneScript (Piscataway, NJ, USA) at >95% purity. The antibody against HMGCoAR was bought from Abcam (Cambridge, UK). Phenylmethanesulfonyl fluoride (PMSF), Na-orthovanadate inhibitors, and the antibodies against rabbit Ig-horseradish peroxidase (HRP), mouse Ig-HRP, and SREBP-2 (which recognizes epitope located in a region between 833–1141 and bands at about 132 kDa) were purchased from Santa Cruz Biotechnology Inc. (Santa Cruz, CA, USA). The antibodies against hepatocyte nuclear factor 1-alpha (HNF1-alpha) and PCSK9 were bought from GeneTex (Irvine, CA, USA). The inhibitor cocktail Complete Midi was from Roche (Basel, Switzerland). Mini protean TGX pre-cast gel 7.5% and Mini nitrocellulose Transfer Packs were purchased from BioRad (Hercules, CA, USA). 

### 2.2. Caco-2 Cell Culture and Differentiation

Human intestinal Caco-2 cells, obtained from INSERM (Paris, France), where cultured according to a published protocol [14]. For differentiation, they were seeded on polycarbonate filters, 12 mm diameter, 0.4 µm pore diameter (Transwell, Corning Inc., Lowell, MA, US) at a 3.5 × 10^5^ cells/cm^2^ density in complete medium supplemented with 10% FBS in both AP and BL compartments for 2 d to allow the formation of a confluent cell monolayer. Starting from day three after seeding, cells were transferred to FBS-free medium in both compartments, supplemented with ITS (final concentration 10 mg/L insulin (I), 5.5 mg/L transferrin (T), 6.7 μg/L sodium selenite (S); GIBCO-Invitrogen, San Giuliano Milanese, Italy) only in the basolateral (BL) compartment, and allowed to differentiate for 18–21 days with regular medium changes three times weekly [15].

### 2.3. Cell Monolayers Integrity Evaluation

The transepithelial electrical resistance (TEER) of differentiated Caco-2 cells was measured at 37 °C using the voltmeter apparatus Millicell (Millipore Co., Billerica, MA, USA), immediately before and at the end of the transport experiments. In addition, at the end of transport experiments, cells were incubated from the AP side with 1 mM phenol-red in PBS containing Ca^++^ (0.9 mM) and Mg^++^ (0.5 mM) for 1 h at 37 °C, to monitor the paracellular permeability of the cell monolayer. The BL solutions were then collected and NaOH (70 µL, 0.1 N) was added before reading the absorbance at 560 nm by a microplate reader Synergy H1 from Biotek (Winooski, VT, USA). Phenol-red passage was quantified using a standard phenol-red curve. Only filters showing TEER values and phenol red passages similar to untreated control cells were considered for peptide transport analysis.

### 2.4. Trans-Epithelial Transport Experiments

Prior to experiments, the cell monolayer integrity and differentiation were checked by TEER measurement as described in detail above. Peptide trans-epithelial passage was assayed in differentiated Caco-2 cells in transport buffer solution (137 mM NaCl, 5.36 mM KCl, 1.26 mM CaCl_2_, and 1.1 mM MgCl_2_, 5.5 mM glucose) according to previously described conditions. In order to reproduce the pH conditions existing in vivo in the small intestinal mucosa, the apical (AP) solutions were maintained at pH 6.0 (buffered with 10 mM morpholinoethane sulfonic acid), and the basolateral (BL)solutions were maintained at pH 7.4 (buffered with 10 mM *N*-2-hydroxyethylpiperazine-*N*-4-butanesulfonic acid). Prior to transport experiments, cells were washed twice with 500 µL PBS containing Ca^++^ and Mg^++^. Peptide transportation by mature Caco-2 cells was assayed by loading the AP compartment with P5 alone, in mixture with YDFYPSSTKDQQS (P3) and LTFPGSDAD (P7), and/or P5-met (500 μM) in the AP transport solution (500 µL) and the BL compartment with the BL transport solution (700 µL). The plates were incubated at 37 °C and the BL solutions were collected at different time points (i.e., 15, 30, 60, 90, and 120 min) and replaced with fresh solutions pre-warmed at 37 °C. All BL and AP solutions collected at the end of the transport experiment were stored at −80 °C prior to analysis. Three independent transport experiments were performed, each in duplicate. In order to assess the involvement of transcytotic process in peptides passage, parallel transport experiments were performed in the presence of 500 nM wortmannin in both the AP and BL compartment, over 60 min incubation time.

### 2.5. LC-MS/MS Operating Conditions

The medium collected at the end of transport experiments from AP and BL chambers (500 µL and 700 µL, respectively) were freeze-dried and residues were solubilized in HPLC water (100 µL). Samples were desalted with C18 resin ZipTip by using 80% ACN, 0.1% FA as eluent (Millipore Corporation, Bedford, MA, USA). Each sample was lyophilized under speed-vacuum for 5 h at 30°C and re-dissolved in 50 μL (0.1% formic acid), before MS analysis. Purified BL samples were analyzed on a SL IT mass spectrometer interfaced with a HPLC- Chip Cube source (Agilent Technologies, Palo Alto, CA, USA). Data were processed with MSD Trap control 4.2, and Data analysis 4.2 version (Agilent Technologies, Palo Alto, CA, USA). The chromatographic separation was performed using a 1200 HPLC system equipped with a binary pump. The peptide enrichment was performed on a 160 nL enrichment column (Zorbax 300SB-C18, 5 μm pore size, Agilent Technologies Italia SpA, Milan, Italy), followed by separation on a 150 mm × 75 μm analytical column packed (Zorbax300SB-C18, 5 μm pore size, Agilent Technologies Italia SpA, Milan, Italy). The samples (1 µL), acidified with formic acid, were loaded onto the enrichment column at a flow rate 4 µL/min using isocratic 100% C solvent phase (99% water, 1% ACN and 0.1% formic acid). After clean-up, P5-met was injected into the mass spectrometer at the constant flow rate of 300 nL/min. The LC solvent A was 95% water, 5% ACN, 0.1% formic acid; solvent B was 5% water, 95% ACN, 0.1% formic acid. The nano-pump gradient program was as follows: 5% solvent B (0 min), 70% solvent B (0–8 min), and back to 5% in 2 min. The drying gas temperature was 300 °C, flow rate 3 L/min (nitrogen). Data acquisition occurred in positive ionization mode. Capillary voltage was −1900 V, with endplate offset −500V. Mass spectra were acquired with ICC target 30,000, and maximum accumulation time 150 ms. The LC/MS analysis were performed in multiple reaction monitoring (MRM) mode. Specifically, The monitored MRM transitions of P5-met were from the mono-charged precursor ion [M^+^H]^+^ (m/z 882.43) to product-ions m/z 678.3 and 407.1, respectively.

### 2.6. Calibration Curve for the Quantification of Absorbed P5-Met and Method Validation

The quantitative analysis of P5-met in the BL samples was carried out by the Ion Trap MS in MRM mode, monitoring two of the most intense diagnostic transitions (882.43➔678.3 and 882.43➔407.1), after optimization of the acquisition parameters, such as retention time, MS profile, and MS/MS fragmentation spectrum (Supplementary Materials, Figure S1). A blank was analyzed between samples to ensure the absence of any carryover effect. Seven different concentrations of standard peptide P5-met ranging from 0.1, 0.4, 0.8, 1.5, 3, 5, and 10 μM were analyzed in three technical replicates. To determine the relation between the peak area under the curves and the concentration of peptide, the calibration curve was built by plotting the mean response factor (peak area) against the respective concentrations of P5-met. Then, BL samples were analyzed using the same optimized parameters. Data were processed by Data analysis v.4.2 (Agilent Technologies, Palo Alto, CA, USA). The peak areas of all monitored transitions from P5-met were integrated and used for the quantification.

The analytical method was validated in terms of selectivity, linearity, limit of detection (LOD), limit of quantification (LOQ), accuracy and precision, according to the guidelines for bioanalytical method validation of the Center for Drug Evaluation and Research of the U.S. Food and Drug Administration (Food and Drug Administration 2001). Quality control samples were obtained by spiking peptide P5-met (0.5 μM) in a BL sample from control Caco-2 cells.

### 2.7. HepG2 Cell Culture Conditions and Treatment

The HepG2 cell line was bought from ATCC (HB-8065, ATCC from LGC Standards, Milan, Italy) and was cultured in DMEM high glucose with stable *L*-glutamine, supplemented with 10% FBS, 100 U/mL penicillin, 100 µg/mL streptomycin (complete growth medium) with incubation at 37 °C under 5% CO_2_ atmosphere.

### 2.8. HMGCoAR Aactivity Assay

The experiments were carried out following the manufacturer instructions and optimized protocol [16]. The assay buffer, NADPH, substrate solution, and HMGCoAR were provided in the HMGCoAR Assay Kit (Sigma Aldrich SRL, Milan, Italy). The experiments were carried out following the manufacturer instructions at 37 °C. In particular, each reaction (200 µL) was prepared adding the reagents in the following order: 1× assay buffer, a 10–500 µM doses of P5 and P5-met or vehicle (C), the NADPH (4 µL), the substrate solution (12 µL), and finally the HMGCoAR (catalytic domain) (2 µL). Subsequently, the samples were mixed and the absorbance at 340 nm read by the microplate reader Synergy H1 (Winooski, VT, USA) at time 0 and 10 min. The HMGCoAR-dependent oxidation of NADPH and the inhibition properties of peptides were measured by absorbance reduction, which is directly proportional to enzyme activity.

### 2.9. In Vitro PCSK9-LDLR Binding Assay

Peptides P5 and P5-met (0.1–100 µM) were tested using the in vitro PCSK9-LDLR binding assay (CycLex Co., Nagano, Japan) following the manufacture instructions and conditions already optimized [10]. Briefly, plates are pre-coated with a recombinant LDLR-AB domain containing the binding site of PCSK9. Before starting the assay, tested peptides and/or the vehicle were diluted in the reaction buffer and added in microcentrifuge tubes. Afterwards, the reaction mixtures were added in each well of the microplate and the reaction was started by adding His-tagged PCSK9 solution (3 μL). The microplate was allowed to incubate for 2 h at room temperature (RT) shaking at 300 rpm on an orbital microplate shaker. Subsequently, wells were washed 4 times with the wash buffer. After the last wash, the biotinylated anti-His-tag monoclonal antibody (100 μL) was added and incubated at RT for 1 h shaking at 300 rpm. After incubation, wells were washed for 4 times with wash buffer. After the last wash, 100 μL of HRP-conjugated streptavidin were added and the plate was incubated for 20 min at RT. After incubation, wells were washed 4 times with wash buffer. Finally, the substrate reagent (tetra-methylbenzidine) was added, and the plate was incubated for 10 min at RT shaking at ca. 300 rpm. The reaction was stopped with 2.0 M sulfuric acid and the absorbance at 450 nm was measured using the Synergy H1 fluorescent plate reader (Winooski, VT, USA).

### 2.10. In-Cell Western (ICW) Assay

For the experiments, a total of 3 × 10^4^ HepG2 cells/well were seeded in 96-well plates. The following day, cells were washed with PBS and then starved overnight (O/N) in DMEM without FBS and antibiotics. After starvation, HepG2 cells were treated with 4.0 μg/mL PCSK9-WT and 4.0 μg/mL PCSK9 + peptides P5 and/or P5-met (50 µM) and vehicle (H_2_O) for 2 h at 37 °C under 5% CO_2_ atmosphere. Subsequently, they were fixed in 4% paraformaldehyde for 20 min at room temperature (RT). Cells were washed 5 times with 100 µL of PBS/well (each wash was for 5 min at RT) and the endogenous peroxides activity quenched adding 3% H_2_O_2_ for 20 min at RT. Non-specific sites were blocked with 100 µL/well of 5% bovine serum albumin (BSA, Sigma) in PBS for 1.5 h at RT. LDLR primary antibody solution (1:3000 in 5% BSA in PBS, 25 µL/well) was incubated O/N at +4 °C. Subsequently, the primary antibody solution was discarded and each sample was washed 5 times with 100 µL/well of PBS (each wash was for 5 min at RT). Goat anti-rabbit Ig-HRP secondary antibody solution (Santa Cruz) (1:6000 in 5% BSA in PBS, 50 µL/well), was added and incubated 1 h at RT. The secondary antibody solution was washed 5 times with 100 µL/well of PBS (each wash for 5 min at RT). Freshly prepared TMB substrate (Pierce, 100 µL/well) was added and the plate was incubated at RT until desired color was developed. The reaction was stopped with 2 M H_2_SO_4_ and then the absorbance at 450 nm was measured using the microplate reader Synergy H1 (Winooski, VT, USA). After the read, cells were stained by adding 1× Janus Green stain, incubating for 5 min at RT. The dye was removed and the sample washed 5 times with water. Afterward 100 µL 0.5 M HCl for well were added and incubated for 10 min. After 10 seconds shaking, the OD at 595 nm was measured using the microplate reader Synergy H1 (Winooski, VT, USA).

### 2.11. Fluorescent LDL Uptake

HepG2 cells (3 × 10^4^/well) were seeded in 96-well plates and kept in complete growth medium for 2 days before treatment. The third day, cells were washed with PBS and then starved overnight (O/N) in DMEM without FBS and antibiotics. After starvation, they were treated with 4.0 μg/mL PCSK9 and 4.0 μg/mL PCSK9 + P5 and P5-met peptides (50.0 µM), and vehicle (H_2_O) for 2 h with at 37 °C under 5% CO_2_ atmosphere. At the end of the treatment, the culture medium was replaced with 50 μL/well LDL-DyLight™ 550 working solution (Cayman Chemical Company, Ann Arbor, MI, USA) prepared in DMEM without FBS and antibiotics. The cells were additionally incubated for 2 h at 37 °C and then the culture medium was aspirated and replaced with PBS (100 μL/well). The degree of LDL uptake was measured using the Synergy H1 fluorescent plate reader (Winooski, VT, USA) (excitation and emission wavelengths 540 and 570 nm, respectively). Fluorescent LDL-uptake was finally assessed following optimized protocol [12].

### 2.12. Western Blot Analysis

Immunoblotting experiments were performed using optimized protocol [12]. A total of 1.5 × 10^5^ HepG2 cells/well (24-well plate) were treated with 50 μM of P5 and P5-met for 24 h. After each treatment, the supernatants were collected and stored at −20°C; cells were scraped in 40 µL ice-cold lysis buffer (RIPA buffer + inhibitor cocktail  +  1:100 PMSF  +  1:100 Na-orthovanadate + 1:1000 β-mercaptoethanol) and transferred in ice-cold microcentrifuge tubes. After centrifugation at 13,300 g for 15 min at 4 °C, the supernatants were recovered and transferred into new ice-cold tubes. Total proteins were quantified by the Bradford’s method and 50 μg of total proteins loaded on a pre-cast 7.5% Sodium Dodecyl Sulfate-Polyacrylamide (SDS-PAGE) gel at 130 V for 45 min. Subsequently, the gel was pre-equilibrated in H_2_O for 5 min at room temperature (RT) and transferred to a nitrocellulose membrane (Mini nitrocellulose Transfer Packs,) using a Trans-Blot Turbo at 1.3 A, 25 V for 7 min. Target proteins, on milk or BSA blocked membrane, were detected by primary antibodies as follows: anti-SREBP-2, anti-LDLR, anti-HMGCoAR, anti-PCSK9, anti HNF1-α and anti-β-actin. Secondary antibodies conjugated with HRP and a chemiluminescent reagent were used to visualize target proteins and their signal was quantified using the Image Lab Software (Biorad, Hercules, CA, USA). The internal control β-actin was used to normalize loading variations.

### 2.13. Quantification of PCSK9 Secreted by HepG2 Cells through ELISA

The supernatants collected from treated HepG2 cells (50.0 μM of P5 and/or P5-met) were centrifuged at 600 × g for 10 min at 4 °C and ELISA assay performed using protocol already optimized [12]. They were recovered and diluted to the ratio 1:10 with DMEM in a new ice-cold tube. PCSK9 was quantified by ELISA (R&D System, Minneapolis, MN, USA). Briefly, the experiments were carried out at 37 °C, following the manufacturer’s instructions. Before starting the assay, human PCSK9 standard curve (20.0, 10.0, 5.0, 2.5, 1.25, and 0.625 ng/mL) was prepared by serial dilutions from a 40 ng/mL stock. 100 µL of the Assay Diluent RD1-9 (provided into the kit) were placed in each well, before adding the standards and the samples (50 µL) and incubating the ELISA plate for 2 h at RT. Subsequently, wells were washed 4 times with the wash buffer, and 200 µL of human PCSK9-conjugate (HRP-labelled anti-PCSK9) was added to each well for 2 h at RT. Following aspiration, wells was washed 4 times with the kit wash buffer. After the last wash, 200 µL of substrate solution were added to the wells and allowed to incubate for 30 min at RT. The reaction was stopped with 50 µL of the stop solution (2 M sulfuric acid) and the absorbance at 450 nm was measured using Synergy H1 microplate (Winooski, VT, USA).

### 2.14. Molecular Modeling

The PCSK9/P5-met model was built starting from the coordinates of the PCSK9/P5 complex model reported by us [10]. Here, the first two residues (residues LI) of peptide P5 were manually removed from the PCSK9/P5 complex model, by means of the molecular modeling tools available in Maestro software (Schrödinger Inc, Mannheim, Germany.). Then, the resulting complex model was energy minimized and equilibrated through three MD simulations replicas (each lasting more than 300 ns) utilizing the pmemd.cuda module of AMBER 2017 package [17]. In the production runs, the computational protocol applied in our previous studies [18,19] was applied. In particular, the ff14SB AMBER force field [20] was used for the protein, while the TIP3P model [21] was used to explicitly represent water molecules (about 25,000). Then, after the addition of the sodium ions needed to neutralize the overall charge of the simulation system, the MD trajectories acquired in the production runs were examined by visual inspection with VMD [22], ensuring that the thermalization did not cause any structural distortion. Finally, the three MD replicas’ trajectory frames were collected in order to cluster the conformations assumed by the small peptide backbone atoms bound on the PCSK9 surface. The cluster analysis was performed using the cpptraj module of AMBER17 [17]. By this, the MD frames were divided into clusters by the complete average linkage algorithm, and the PCSK9/P5-met complex conformations with the lowest root mean square deviation (RMSD) to the cluster centers (the structures representative of the cluster, SRC), were acquired and visually inspected. MM-GBSA calculations were performed on the most populated cluster of P5-met conformations, the MMPBSA.py module of AMBER17 [17] package was used to this aim. The computational details and applied parameters of these calculations were the ones reported on our previous paper [19].

### 2.15. Circular Dichroism (CD) Spectroscopy

Circular dichroism (CD) spectra were recorded in continuous scanning mode (190–300 nm) at 25 °C using a Jasco J-810 (Jasco Corp., Tokyo, Japan) spectropolarimeter. All spectra were collected using a 1 mm path-length quartz cell and averaged over three accumulations (speed: 10 nm min^−1^). A reference spectrum of distilled water was recorded and subtracted from each spectrum. 

### 2.16. Statistical Analysis

All the data sets were checked for normal distribution by D’Agostino and Pearson test. Since they are all normally disturbed with *p*-values < 0.05, we proceeded with statistical analyses by One-Way ANOVA followed by Dunnett’s and Tukey’s post-hoc tests and using Graphpad Prism 9 (San Diego, CA, USA). Values were reported as means ± S.D.; *p*-values < 0.05 were considered to be significant.

## 3. Results

### 3.1. Intestinal Transport of P5 Alone or in Combination with Other Peptides across Caco-2 Cells

**Figure 1:**
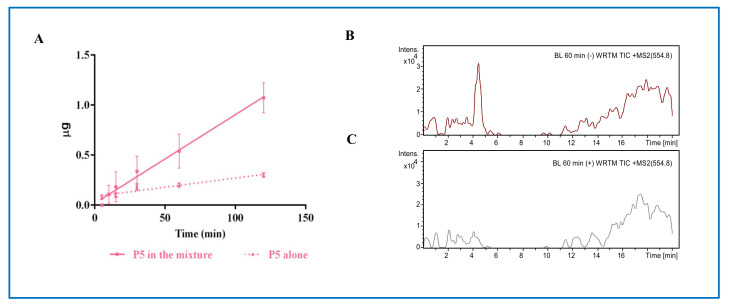
Transport of P5 across differentiated Caco-2 cells. (**A**) Quantification of P5 in the basolateral (BL) compartment as a function of time; pink dashed line: P5 alone; pink line: in mixture. Data represent the mean ± SD of three independent experiments performed in triplicate. (**B**) HPLC-Chip MS of BL compartment at time 60 min: total ion current (TIC) of [M + 2H]^2+^ *m*/*z* 554.8 without wortmannin, and (**C**) with wortmannin.

Recently, we have demonstrated that the intestinal transport of a peptide is highly influenced by the presence of other peptides [13]. For this reason, the kinetics of the transport of P5 was investigated in two different conditions, i.e., either when it was alone or in a mixture with P3 (YDFYPSSTKDQQS) and P7 (LTFPGSAED), two lupin peptides that had already been shown to be transported in the same model system [9,13]. Each peptide was added in the AP compartment at the final concentration of 500 μM. As shown in Figure 1A, in both conditions, P5 was linearly transported across the Caco-2 cells monolayer as a function of time. When it was alone, the rate of absorption was 16.3 ± 0.3 nmoles/(mL × min) (*R^2^* 0.999), whereas in the mixture the rate was 80.3 ± 0.4 nmoles/(mL × min) (*R^2^* 0.988) (Figure 1A). Moreover, at the end of the incubation (2 h), the amount of P5 in the BL compartment was about 3.5-fold higher when it was tested in mixture (1.1 ± 0.2 μg, equal to 0.99 nmoles) than when it was tested alone (0.3 ± 0.02 μg, equal to 0.271 nmoles). Both evidences suggest that the presence of other peptides favored the transport possibly by increasing the stability of P5 and impairing its degradation.

Food derived peptides may be transported across the intestinal brush-border membrane into the bloodstream via one or more of the following routes: (i) peptide transport 1 (PepT1)-mediated route, (ii) paracellular route via tight junctions (TJs), (iii) transcytosis route, and (iv) passive transcellular diffusion [23]. Peptide size, charge, hydrophobicity, and degradation due to the action of peptidases are among the main factors influencing the absorption through one or more of these routes. In general, short peptides, such as dipeptides and tripeptides, are preferentially transported by PepT1, due to its high-capacity, low-affinity, and high expression in intestinal epithelium [24], whereas highly hydrophobic peptides are transported by simple passive transcellular diffusion or by transcytosis [25]. Since P5 is a decapeptide with a net charge equal to −1 and a hydrophobicity of +17.79 kcal/mol, it may be hypothesized that it might be preferentially transported by passive transcellular diffusion or by transcytosis. Whereas it is difficult to assess the transport via the former route, due to the lack of passive diffusion regulators, wortmannin can be used as transcytosis inhibitor to investigate the latter route. Therefore, specific experiments in the presence of wortmannin were performed in order to assess the mechanism of transport of peptide P5 across the Caco-2 cell layer. As shown in Figure 1B,C, wortmannin significantly impairs the transport of P5, strongly suggesting that P5 is mainly transported by the transcytotic route.

### 3.2. Analysis of the Metabolites of P5 Produced by Caco-2 Cells

Mature enterocytes develop microvilli that function as the primary surface of nutrient absorption in the gastrointestinal tract. Their membrane is packed with enzymes that favor the breakdown of complex nutrients into simpler compounds that are more easily absorbed. The dynamic equilibrium between bioactive peptide degradation and transport is crucially important from a physiological point of view. Possessing a wide range of membrane peptidases naturally expressed by the AP side of enterocytes, including DPP-IV and ACE [26,27,28], the differentiated Caco-2 model is also a reliable tool for investigating the proteolytic activity of the brush border barrier. 

Under the hypothesis that during the transport experiments P5 may also be metabolized by the enzymes expressed on the AP surface of the Caco-2 cells, it was decided to look for possible metabolites through mass spectrometry analysis of the AP solutions. Indeed, P5 was dynamically metabolized in two main breakdown fragments (Table 1): ILPKHSDAD (P5-frag, m/z 995.51), deriving from the cleavage of a leucine from the N-terminal of the parent peptide, and LPHKSDAD (P5-met, m/z 882.43), formally deriving from the cleavage of a leucine-isoleucine fragment from the N-terminal of P5. An aminopeptidase, such as leucine aminopeptidase (LAP), may catalyze the hydrolysis of the leucine residue at the N-terminus of the parent peptide generating P5-frag and then the N-terminal isoleucine may be further cleaved to generate P5-met. However, P5-met may also derive from the direct cleavage of a leucine-isoleucine fragment from P5. Instead, P5 does not seem to be susceptible to the action of endopeptidases, such DPP-IV.

Interestingly, whereas P5-met was abundant and was produced in both conditions, P5-frag was detected only when P5 was tested in combination with P3 and P7 and, based on the spectral intensity, was a minor metabolite. This suggests that the transformation of P5-frag into P5-met may be such a fast process that only the concomitant presence of very easily cleavable peptides (like P7) permitted its detection, by protecting it from degradation. However, we cannot exclude that other smaller fragments, such as tripeptides and dipeptides, were also generated in these conditions, since they are intrinsically difficult to detect.

### 3.3. Characterization of the Transport of P5-Met Across the Caco-2 Cell Monolayer

**Figure 2:**
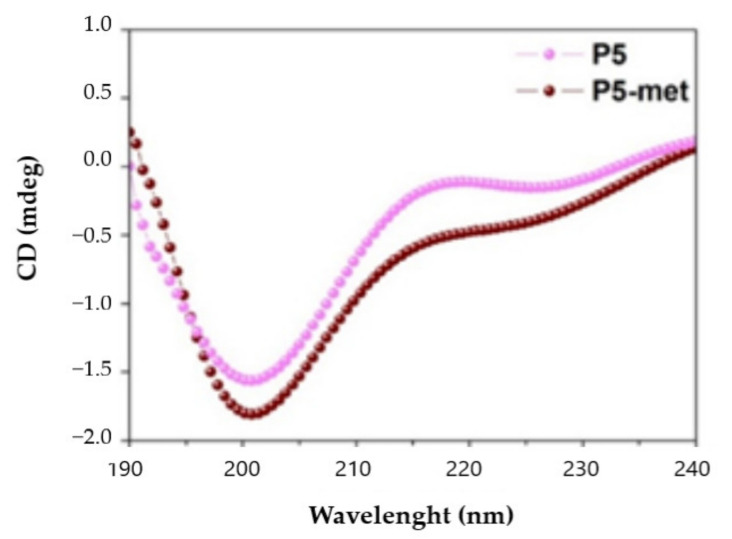
Circular dichroism (CD) spectra of P5 and P5-met registered in the range of 190–240 nm.

Since the octapeptide P5-met was the most abundant and the smallest metabolite, it was decided to synthetize it in order to obtain structural, functional, and biological characterization. To compare the secondary structure of P5-met and the parent compound P5, the CD spectra in the far-UV region of 190–240 nm were recorded. One negative peak was observed at 200 nm, suggesting that both peptides have a random coil conformation (Figure 2) and confirming the three-dimensional structure of P5 at the end of MD simulations and subsequent energy minimization [10].

**Figure 3:**
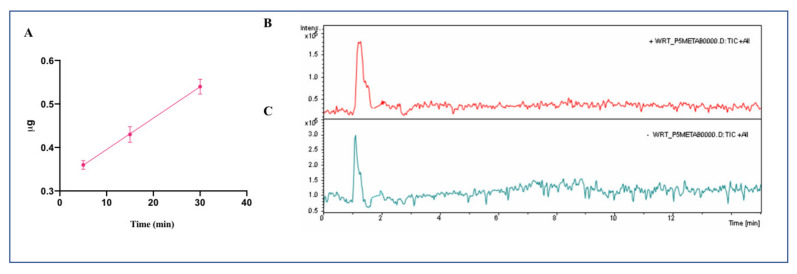
Transport of P5-met across differentiated Caco-2 cells. (**A**) Quantification of P5-met in the BL compartment as a function of time. Data represent the mean ± SD of three independent experiments performed in triplicate. The accuracy of analytical was d higher than 95%. LOQ was 0.10 µg whereas LOD was detected equal to 0.09 µg. (**B**) HPLC-Chip MS of BL compartment at time 60 min: TIC of P5-met without wortmannin, and (**C**) with wortmannin.

Afterward, trans-epithelial transport experiments were performed using differentiated Caco-2 cells (Figure 3): the rate of transport of P5-met (incubated alone in the AP compartment at the concentration of 500 μM) up to 30 min was 81.7 nmoles ± 0.3 ng/(mL × min) (*R^2^* 0.99). A decline in transport rate was observed after 60 min (data not shown) probably due to a decline of P5-met concentration in the donor AP compartment, caused by metabolism. The concentration of P5-met in the BL compartment after 30 min (0.54 ± 0.02 µg, equal to 0.613 nmoles) (Figure 3) was much higher than that of the parent peptide tested alone after 60 min (0.20 ± 0.02 µg, equal to 0.227 nmoles) (Figure 1), suggesting that P5-met is either efficiently transported or poorly metabolized by Caco-2 cells or both. Additional experiments showed that P5-met is transported also in the presence of wortmannin (Figure 3B), indicating that the mechanism of transport may involve a passive diffusion mechanism or passage through the paracellular route. It is important to underline, however, that dedicated experiments would be required for a complete characterization of the transport mechanism of P5-met. Interestingly, similar results have been obtained in transport experiments performed with LTFPG, a metabolite of peptide P7 [13].

### 3.4. P5-Met Modulates the Hepatic LDLR Pathway through the Inhibition of HMGCoAR Activity

**Figure 4:**
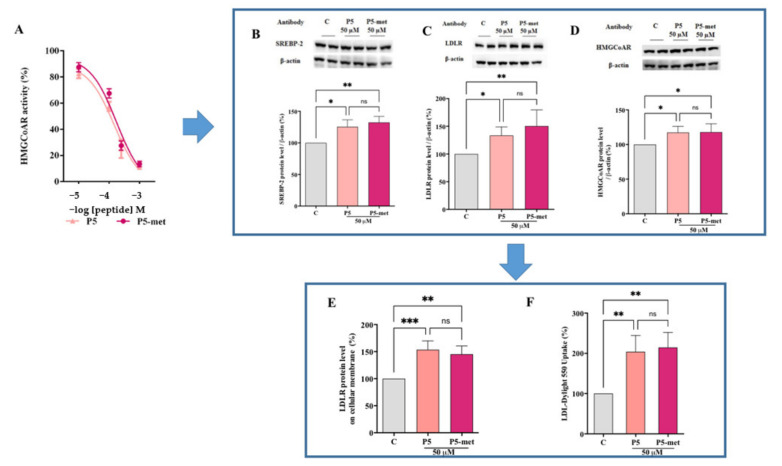
Modulation of low-density lipoprotein receptor (LDLR) pathway in HepG2 cells treated with P5 and P5-met. (**A**) In vitro inhibition of the HMGCoAR activity with IC_50_ values equal to 147.2 and 175.3 µM, respectively. (**B**) After the treatment of HepG2 cells with P5 and P5-met, the SREBP-2 protein level was increased, as well as (**C**) the LDLR and (**D**) the HMGCoAR protein levels, and (**E**) the LDLR localized on the surface of hepatic cell. (**F**) Enhancement of functional ability of hepatic cells to uptake LDL from the extracellular environment. Data points represent the averages ± SD of four independent experiments performed in duplicate. C vs. P5 and P5-met samples were analyzed by One-Way ANOVA followed by Dunnett’s test; (*) *p* <0.05; (**) *p* < 0.01 (***) *p* < 0.0001. C: control sample; ns: not significant.

A biochemical investigation was carried out for assessing the ability of P5-met to modulate in vitro HMGCoAR activity. P5-met was active as HMGCoAR inhibitor with an IC_50_ of 175.3 µM (Figure 4A), similarly to the parent peptide, which displayed an IC_50_ of 147.2 µM, whereas the positive control pravastatin reduced the enzyme activity by 82.0% at 1.0 µM, as indicated in the Supplementary Materials (Figure S2). Based on these results, further experiments aimed at comparing the ability of P5-met and P5 to modulate the LDLR and PCSK9 pathways on HepG2 cells were carried out at the fixed concentration of 50 µM, which was the same concentration already used for testing peptide P5 on the same cellular system [12], although different from that absorbed by Caco-2 cells after the incubation of 500 µM in the AP side. P5-met and P5 induced an up-regulation of the protein level of the SREBP-2 transcription factor to 130.4 ± 16.4% and 125.7 ± 16% (*p* < 0.05), respectively, versus the control (Figure 4B). As a consequence, the LDLR protein levels were increased up to 150.4 ± 29.2% and 133.4 ± 15.5% (*p* < 0.001), respectively (Figure 4C), and the HMGCoAR protein levels up to 117.9 ± 12.1% and 117.3 ± 9.1% (*p* < 0.05), respectively (Figure 4D). In agreement with these results, P5-met and P5 induced an increased expression of the LDLR localized on the cellular membranes of HepG2 cells by 145.2 ±15.2% and 153.6 ± 16.4% (*p* < 0.0001), respectively, versus the control (Figure 4E). The overall up-regulation of the LDLR protein levels, led to an increased functional ability of HepG2 cells to absorb extracellular LDL up to 181.2 ± 40.1% versus the control after treatment with P5-met and by 174.3 ± 32.3% after treatment with P5 (Figure 4F).

### 3.5. P5-Met Modulates the Hepatic PCSK9 Pathway and Impairs the PPI between PCSK9 and the LDLR

**Figure 5:**
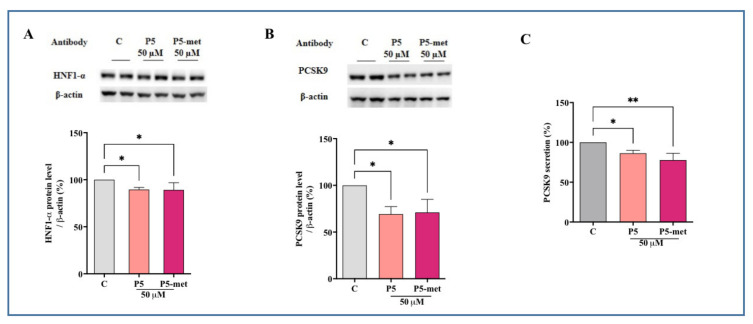
Modulation of PCSK9 pathway in HepG2 cells treated with P5 and P5-met. (**A**) Effects on the HNF1-α protein level; (**B**) effects on the PCSK9 protein levels; (**C**) effects on mature PCSK9 secretion. Data points represent the averages ± SD of six independent experiments performed in duplicate. C versus P5 and P5-met samples were analyzed by One-Way ANOVA followed by Dunnett’s test; (*) *p* < 0.05, (**) *p* < 0.01. C: control sample.

PCSK9 is a secreted protein expressed in many organs, such as liver, kidney, and intestine, which is able to bind the LDLR expressed on the surface of the hepatocytes [29]. PCSK9-LDLR binding activates the receptor catabolism leading to degradation of the hepatic LDLR. Thus, PCSK9 inhibition and/or modulation are considered promising strategies for the development of new hypocholesterolemic drugs [30]. Interestingly, PCSK9 and LDLR are co-regulated by SREBP-2, since both proteins contain functional sterol regulatory elements (SREs) in their promoters that respond to changes in intracellular cholesterol levels through the activation of the SREBP pathway [31,32]. However, since the HNF-1α binding site is unique to the PCSK9 promoter and is not present in the LDLR promoter, the modulation of the PCSK9 transcription through HNF-1α does not affect the LDLR gene expression. Thus, the co-regulation of PCSK9 from LDLR and other SREBP target genes is disconnected by the HNF-1α binding site [33]. Similarly to P5, P5-met decreases the hepatic PCSK9 production and extra cellular secretion through the downregulation of the HNF-1α protein content in HepG2 cells (Figure 5A–C). More in detail, P5-met decreases the HNF-1α protein by 10.8 ± 3.5% and P5 by 7.7 ± 1.5% (*p* < 0.01; Figure 5A). This slight, but significant reduction leads to a decrease of PCSK9 by 29 ± 8.1% and 31 ± 4.6% for P5-met and P5, respectively (*p* < 0.05; Figure 5B). In agreement, both P5-met and P5 are also able to decrease the secretion of mature PCSK9 by 22.2 ± 4.9% and 12.3 ± 1.6%, respectively (*p* < 0.05; Figure 5C).

**Figure 6:**
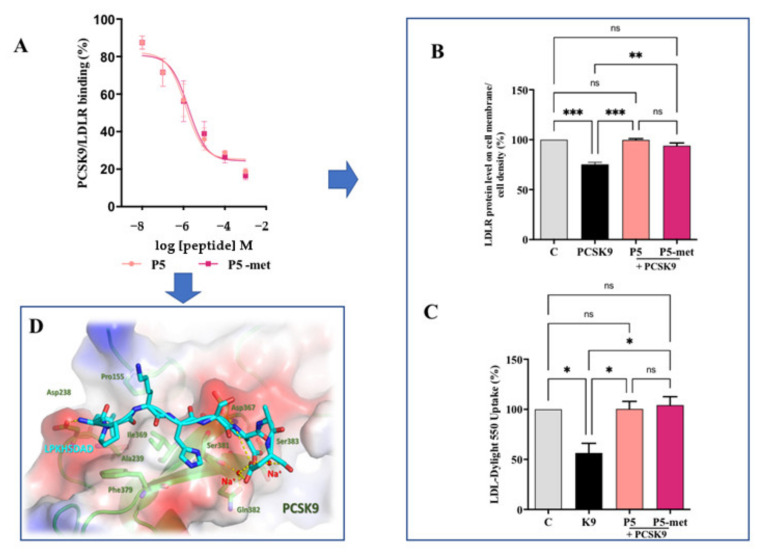
Inhibition of the PPI between PCSK9 and LDLR. (**A**) Impairment of the protein–protein interaction between PCSK9 and LDLR. (**B**) The treatment of HepG2 cells with PCSK9 (4 µg/mL) reduced active LDLR protein levels localized on the surface of cells, which were restored by P5 or P5-met (50 µM). (**C**) The decreased functional ability of HepG2 cells to absorb LDL from the extracellular space observed after incubation with PCSK9 (4 µg/mL) is improved after treatment with both peptides. (**D**) Hypothetical binding mode of P5-met in the LDLR binding site located on the surface of PCSK9. The data points represent the averages ± SD of four independent experiments performed in duplicate. Data were analyzed by One-Way ANOVA followed by Tukey’s post-hoc test; (*) *p* < 0.05, (**) *p* < 0.01, and (***) *p* < 0.0001. C: control sample; ns: not significant.

Aiming at the evaluation of the multi-target inhibitory ability of P5-met, experiments were performed in order to assess its ability to directly impair the PCSK9-LDLR PPI. Results indicate that P5-met reduces PCSK9-LDLR binding with a dose-response trend and an IC_50_ of 1.7 μM (Figure 6A), which is similar to the IC_50_ of P5 (1.3 μM) tested in parallel. This last result confirms P5 activity as an inhibitor of the PCSK9-LDLR PPI observed in a previous paper [10]. The effects on the modulation of the LDLR localized on the HepG2 cell surface were investigated using an ICW assay in the presence of PCSK9 [34]. Findings indicate that the LDLR protein levels decrease in the presence of PCSK9 alone by 24.7 ± 1.9% (*p* < 0.001) versus the control cells, and that P5 and P5-met (50 µM) are able to significantly increase the LDLR protein levels when co-incubated with PCK9 (*p* < 0.0001). In particular, peptide P5 restored the LDLR level up to 99.3 ± 1.8%, whereas peptide P5-met up to 90.1 ± 0.1% (Figure 6B). Finally, functional experiments were carried out for assessing the ability of each peptide to modulate the capacity of HepG2 cells to uptake extracellular LDL, treating HepG2 cells with PCSK9 alone or in the presence of each peptide (50 µM). After treatment with PCSK9 alone, the ability of HepG2 cells to uptake fluorescent LDL was reduced by 43.5 ± 9.7% (*p* < 0.05) versus untreated cells (Figure 6C), and the treatment with P5 and P5-met reversed this effect up to 100.4 ± 7.5% and 104.1 ± 8.5% (*p* < 0.05), respectively (Figure 6D).

To get a deeper inside on these phenomena, it was decided to investigate the interaction of P5-met with PCSK9 through a dedicated computational study. Notably, the 3D structure of the PCSK9/P5-met complex was modeled and refined following the procedure described in the Section 2.14. Essentially, the simulations system (enzyme, small peptide, ions, and water) was equilibrated and optimized by means of three 300 ns-long MD simulations replicas. The attained trajectories showed that the small peptide displayed diverse binding modes over the simulation time, although it remained firmly anchored on the PCSK9 surface. Then, a cluster analysis of the trajectory frames was accomplished in order to establish which was the most preferred conformation of the small peptide and to acquire major details on the PCSK9 area involved in the interaction with P5-met.

The results of these calculations showed that the small peptide backbone atoms, in the 84% of frames, was bound on PCSK9 area responsible for the interaction with the EGFA domain of LDLR, confirming that P5-met is a PCSK9-LDLR PPI inhibitor. Moreover, the visual inspection of the P5-met conformation displaying the lowest binding free energy (calculated by MM-GBSA approach) permitted to acquire details on the small peptide hypothetical binding mode (Figure 6D). In our hypotheses, the side chain of the Leu1 was inserted in the hydrophobic pocket shaped by the PCSK9 residues Pro155, Ile369, Ala239, and Phe379. Additionally, an H-bond network stabilized P5-met on the PCSK9 surface, these interactions were shaped by: 1) the side chain of the small peptide His4 and the backbone of PCSK9-Ser381, 2) the peptide charged N-term and the PCSK9-Asp238 side chain (this bond additionally enforced by a salt bridge), 3) the backbone NH of Asp6 with the side chain of PCSK9-Asp367, 4) the side chain Asp6 with the one of PCSK9-Ser383. Finally, it is useful to note that two sodium ions (needed to neutralize the total charge of the simulation system) were greatly involved in the stabilization of the binding mode of P5-met. In fact, the negatively charged peptide C-term and the side chains of the P5-met-Asp6 and -Asp8 interacted with the side chain of PCSK9-Gln382 and the backbone atoms of PCSK9-Ser381 and -Ser383. Both electrostatic interactions were bridged by the presence of two positively charged sodium ions (Figure 6D).

## 4. Discussion

In the field of food bioactive peptides, some activities have been investigated much more extensively than others, i.e., the angiotensin converting enzyme (ACE) and DPP-IV inhibitory activity [35,36]. Hence, many hypotensive and antidiabetic peptides have been identified from several different food matrices. On the contrary, only scarce and incomplete information are available about hypocholesterolemic peptides. In this panorama, lupin peptide P5 represents a unique case of multi-target biological activity. In fact, P5 is able to inhibit both HMGCoAR and PCSK9 activities, showing a multi-target behavior. Both enzymes are among the main targets for the treatment of the hypercholesterolemia and the prevention of cardiovascular disease [37]. Some peptides able to inhibit HMGCoAR have been identified from amaranth [38], soybean [39,40], lupin [12], and cowpea [41] proteins. 

On the contrary, PCSK9 is a new target for the prevention and treatment of hypercholesterolemia [42,43], which has been only rarely investigated in the area of food bioactive peptides. In fact, P5 is the unique peptide described so far that is able to inhibit PCSK9. This gap of knowledge on the one hand suggests the need to increase the efforts for identifying new active peptides from different food sources, and on the other indicates that it is crucial to promote the exploitation of the few known active species through in-depth studies regarding, in particular, their bioavailability. Indeed, intestinal metabolism and transport of bioactive peptides still remain relevant issues that need to be addressed in order to overcome the discrepancy observed between in vitro assays and in vivo results and to select good candidates to be translated into practice.

From a physiological point of view, the dynamic equilibrium between the transport and degradation of bioactive peptides is crucially important. In this context, a relevant outcome of this study is the demonstration that the transport across the Caco-2 monolayer is highly affected by the concomitant presence of other peptides. Indeed, the transport is more efficient and its degradation less extensive when P5 is incubated in the presence of peptides P3 and P7. It is important to note, however, that P5 and P7 have completely different behaviors, since the transport rate of P5 tested alone is 1.8 ± 0.3 ng/(mL × min) and in mixture becomes 8.9 ± 0.4 ng/(mL × min), whereas the transport rate of P7 alone is 4.2 ± 0.6 ng/(mL × min) and in mixture becomes 1.98 ± 0.21 ng/(mL × min) [13]. The outcomes on P5 and P7 are therefore divergent, being the transport of P5 favored by the presence of the other peptides, while that of P7 impaired. P5 appears to be less sensitive to the activity of endopeptidases, such as DPP-IV, but more sensitive to the activity of aminopeptidases, which generate two main breakdown fragments, i.e., P5-frag and P5-met. Instead, when tested in mixture, P7 is sensitive not only to the action of aminopeptidases, such as LAP, but also to endopeptidases, such as DPP-IV, leading to the formation of metabolites TFPGSAED and LTFPG, respectively [13].

Another relevant outcome of this study is the information acquired on the mechanism of transport of P5 and P5-met. In fact, although both species are efficiently transported, P5 is mainly transported by transcytosis, whereas P5-met mainly by the paracellular or other passive mechanism.

The biological characterization of P5-met indicates that it retains the multi-target inhibitory activity of the parent peptide on HMGCoAR and PCSK9. In addition, P5-met is also capable to modulate the PCSK9 signaling pathway at the intracellular level, leading to a decrease of mature PCSK9 secretion through the reduction of HNF-1α. More in detail, after the inhibition of HMGCoAR, P5-met leads to the up-regulation of the LDLR pathway, with an increase of LDLR protein levels due to the augmentation of SREBP-2 transcription factor. The molecular modulation of the LDLR-pathway has the consequence of the improvement of the functional ability of HepG2 cells to uptake LDL from the extracellular environment. Notwithstanding the SREBP-2 activation, the HNF-1α protein level reduction leads to a significant decrease of PCSK9 protein level and a subsequent reduction of mature PCSK9 secretion. Indeed, these results highlight the very original hypocholesterolemic mechanism of action of P5 and P5-met that differs from the mechanism of statin. In fact, statins increase PCSK9 expression, which dampens an effective LDL clearing by promoting LDLR degradation [31], thereby counteracting their therapeutic effects. Instead, literature suggests that curcumin [44], from *Curcuma longa,* and berberine [45,46], from plants of the *Berberidaceae* family, display a hypocholesterolemic activity through the reduction of PCSK9 protein levels.

P5 and P5-met are also able to impair the PPI between PCSK9 and the LDLR. The experimental results were confirmed in silico, through the prediction of the binding mode of P5-met in the LDLR binding site located on the PCSK9 target. The inhibition of PCSK9-LDLR by P5-met leads to an efficient restoration of active LDLR protein levels localized on the cellular membrane of hepatocytes co-incubated with PCSK9 and P5 or P5-met versus HepG2 cells incubated with PCSK9 alone. Accordingly, a recovery of the functional capacity of HepG2 cells to uptake extracellular LDL is also observed. In light of these results, we propose a new concept of hypocholesterolemic peptide, based on the modulation of peripheral cholesterol homeostasis rather than simple cholesterol inhibition. In this context, P5 and P5-met are unique and interesting examples of food-derived intrinsically multi-target peptides able to exert complementary effects on the regulation of cholesterol metabolism, which undoubtably opens the route toward a new area of active molecules with cholesterol-lowering properties. Finally, our results suggest that P5 may be a good candidate for further in vivo study.

## Figures and Tables

**Table 1:** Metabolites of P5 identified in the AP compartment of the Caco-2 cell model system at the end of incubation (120 min).

Metabolite Sequence	ID	[M + H]^+^ (Da)	m/z (Da)	Spectral Intensity	Rt(min)	Mixture	Alone
ILPKHSDAD	P5-frag	995.51	332.42	3.88 × 10^6^	2.2	x	n.d.
LPKHSDAD	P5-met	882.43	441.51	1.21 × 10^7^	2.2	x	x

x, detected; n.d., not detected.

## Data Availability

The data presented in this study are available in the results section.

## Supplementary Materials

The following are available online at https://www.mdpi.com/2072-6643/13/3/863/s1, Figure S1. Transport of P5 across differentiated Caco-2 cells, Figure S2. Effect of pravastatin (1.0 µM) on the HMGCoAR activity.
