# Supplementary material for: Trans-Epithelial Transport, Metabolism, and Biological Activity Assessment of the Multi-Target Lupin Peptide LILPKHSDAD (P5) and Its Metabolite LPKHSDAD (P5-Met)
Source: Nutrients. 2021 Mar 5;13(3):863. doi: 10.3390/nu13030863 (PMC8000724; doi:10.3390/nu13030863)
Supplement: Supplementary file 1 [file nutrients-13-00863-s001.pdf]

Supplementary Materials

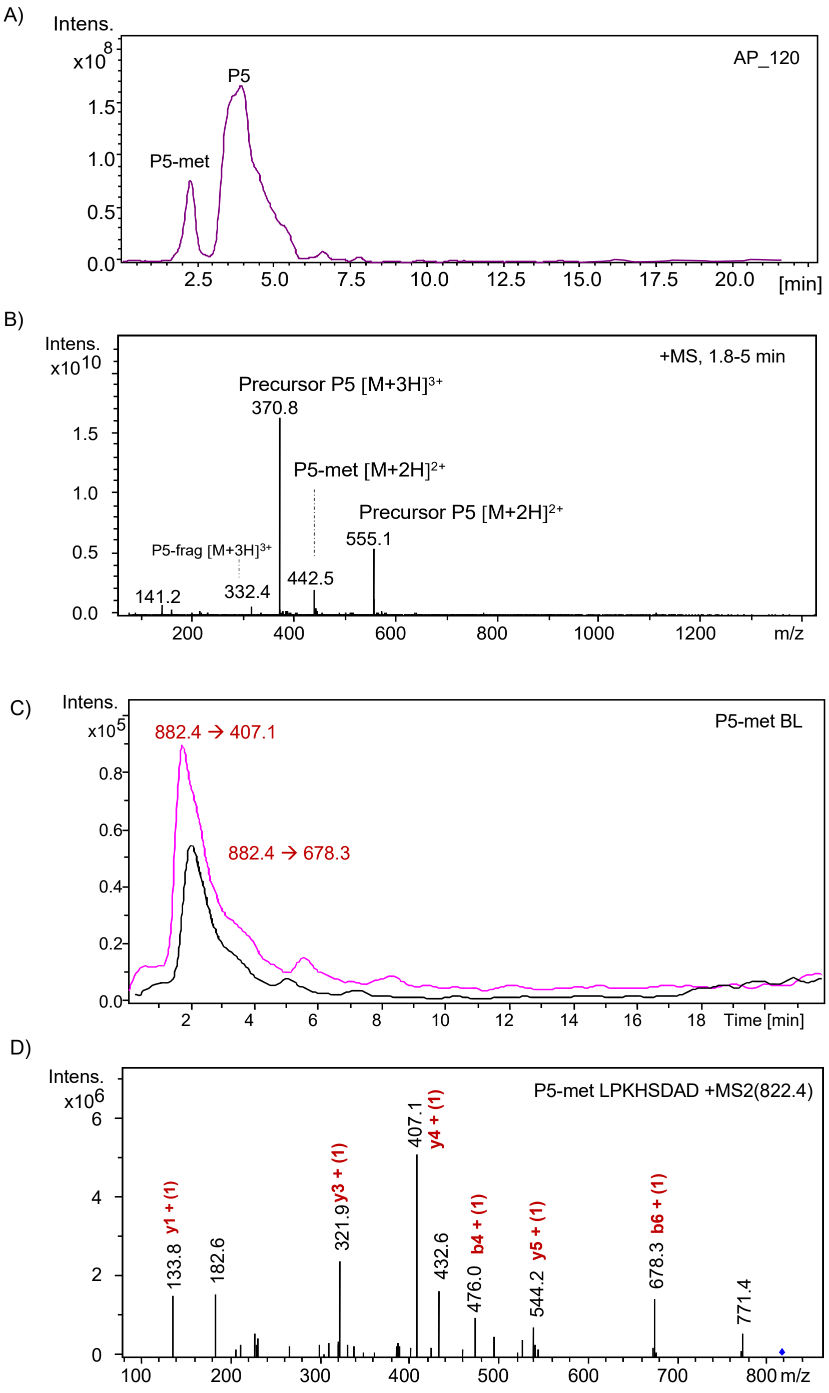

Figure S1. Transport of P5 across differentiated Caco-2 cells. (**A**) TIC spectrum of AP compartment at time 60 min. (**B**) MS spectrum. (**C**) HPLC- MRM of 882.4 → 407.1; 882.4 → 678.3. (**D**) MS/MS spectrum of P5-met LPKHSDAD m/z 882.4 with peptide sequence coverage.

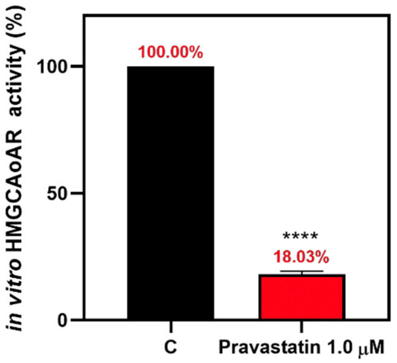

Figure S2. Effect of pravastatin (1.0 μM) on the HMGCoAR activity. **** $p<0.0001$
